# Successful treatment with fludarabine and cyclophosphamide in a VEXAS syndrome patient with associated myelodysplastic syndrome: a case report and systematic review

**DOI:** 10.3389/fonc.2024.1383730

**Published:** 2024-04-11

**Authors:** Polina Bellman, Jesus D. Gonzalez-Lugo, Moazzam Shahzad, Muhammad Kashif Amin, Muhammad Fareed Khalid, Nahid Suleman, Nausheen Ahmed, Anurag K. Singh, Abdulraheem Yacoub, Da Zhang, Joseph P. McGuirk, Muhammad Umair Mushtaq

**Affiliations:** ^1^ Division of Hematologic Malignancies and Cellular Therapeutics, University of Kansas Medical Center, Kansas City, KS, United States; ^2^ Division of Hematology and Oncology, Moffitt Cancer Center, University of South Florida, Tampa, FL, United States; ^3^ Division of Pathology and Laboratory Medicine, University of Kansas Medical Center, Kansas City, KS, United States

**Keywords:** myelodysplastic syndrome, VEXAS syndrome, outcomes, allogeneic hematopoietic stem cell transplantation, fludarabine and cyclophosphamide

## Abstract

Vacuoles, E1 syndrome, X-linked, autoinflammatory, somatic (VEXAS) syndrome is a chronic inflammatory disorder that affects various organ systems. It is associated with hematologic malignancies and is generally refractory to therapies. Allogeneic hematopoietic stem cell transplantation (allo-HSCT) may be considered for selected patients. We report a case wherein systemic and hematological manifestations completely resolved in a patient with VEXAS and associated myelodysplastic syndrome (MDS), following the administration of fludarabine and cyclophosphamide as part of the preparation for allo-HSCT. We conducted a systematic literature review and included 86 patients with VEXAS syndrome and associated MDS. Most cases presented with musculoskeletal involvement (71%) and anemia (72%) with lower-risk MDS. Most patients responded to corticosteroids (CS) but had a recurrence of symptoms with CS taper and were refractory to other immunosuppressive agents. Hypomethylating agents and Janus kinase inhibitors achieved a complete response in some cases. Further research is needed to develop more effective treatment strategies.

## Introduction

Vacuoles, E1 syndrome, X-linked, autoinflammatory, somatic (VEXAS) syndrome is a recently reported pathological entity that presents in late adulthood with an inflammatory syndrome, fevers, cytopenias, dysplastic bone marrow, and characteristic cytoplasmic vacuoles in erythroid and myeloid precursors. It is caused by myeloid-restricted somatic missense mutations in ubiquitin-like modifier activating enzyme 1 (*UBA1*), which is an X-linked gene encoding for the E1 enzyme that initiates ubiquitination of proteins (1). The diagnosis of VEXAS syndrome requires the identification of *UBA1* mutations by deoxyribonucleic acid (DNA) sequencing.

It has been estimated that VEXAS syndrome occurs in 1 out of every 4269 men older than 50 years and 1 in 26238 women older than 50 years (2). Most patients with VEXAS syndrome meet clinical criteria for inflammatory syndromes, such as relapsing polychondritis, Sweet syndrome, polyarteritis nodosa, and giant-cell arteritis, among others. Hematological manifestations commonly include Myelodysplastic Syndromes (MDS) or Plasma Cell Dyscrasias, with few other hematological conditions reported in the literature (3–10).

There is no current standard of care treatment for VEXAS syndrome. The inflammatory features can be treated with corticosteroids, immunosuppressants, and in some cases, hematopoietic stem cell transplant (HSCT). Herein, we present a case of successful treatment of VEXAS syndrome and related myelodysplastic syndrome (MDS), achieving a complete response with fludarabine and cyclophosphamide as part of the conditioning regimen in preparation for HSCT. We also conducted a systematic literature review to summarize current evidence regarding clinical presentation, hematological findings, treatment, and outcomes of VEXAS syndrome with associated MDS.

## Case description

**Figure 1:**
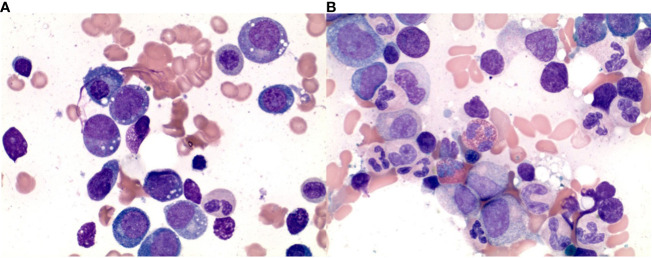
Histopathologic findings in the bone marrow aspirate before and after the treatment. **(A)** Bone marrow aspirate shows vacuolization in the immature myeloid and erythroid series. **(B)** Bone marrow aspirate shows absent vacuolization in the immature myeloid and erythroid series after treatment.

A 66-year-old man with numerous inflammatory manifestations for over 20 years, including recurrent scleritis, relapsing polychondritis, Graves’ disease, bursitis, pyoderma gangrenosum, and leukocytoclastic vasculitis, was evaluated for pancytopenia notable for a white blood cell (WBC) count of 2030/uL with the absolute neutrophil count (ANC) count of 1430/uL, anemia with hemoglobin (Hgb) 9.1 g/dL, mean corpuscular volume of 115 fl and thrombocytopenia with a platelet (PLT) count of 119,000/uL. He had recurrent worsening pancytopenia during inflammatory crises presenting with persistent fatigue, shortness of breath, cough, and fever. On two occasions, his cytopenias worsened to the point of requiring transfusions (initially packed red blood cells (pRBCs), and two months later PLT), though this improved with steroids. For his autoimmune and inflammatory conditions, he had received multiple lines of therapy at an outside institution, including corticosteroids (consistently on prednisone >20 years), methotrexate, dapsone, hydroxychloroquine (several years), rituximab 1 g every two weeks for two courses, adalimumab for a few weeks, tocilizumab (received two monthly infusions but stopped due to worsening symptoms). Several bone marrow biopsies had been performed showing mild dyspoiesis and deletion 20q, increasing in number of cells involved over 10 years from 5% to 24.5%. A bone marrow biopsy was repeated and showed a hypercellular bone marrow with trilineage dyspoiesis, vacuolation of myeloid and erythroid precursor cells, and 1% blasts. Fluorescent *in situ* hybridization (FISH) and cytogenetics showed deletion 20q with otherwise normal male karyotype. Further testing confirmed a pathogenic Met41Thr (c.122 T>C) mutation in the *UBA1* gene consistent with VEXAS Syndrome. Hypomethylating agent was considered but not given due to no increase in blast percentage. He was referred to our clinic for consideration of an allogeneic stem cell transplant. At that time, his Karnofsky Performance Status (KPS) score was 80%, and Eastern Cooperative Oncology Group (ECOG) Performance Status was 1, with no active inflammatory manifestations, apart from intermittent subcutaneous nodules. He remained on 20 mg of prednisone daily and was started on ruxolitinib 5 mg twice daily for systemic symptoms including rash and fever in anticipation of HSCT. His complete blood count was notable for Hgb 7.9 g/dL, PLT 63,000/uL, WBC 6420/uL with ANC 3420/uL. Non-myeloablative conditioning with fludarabine, cyclophosphamide, and total body irradiation (TBI) was planned, followed by HLA-haploidentical peripheral blood stem cell transplantation (PBSCT). After receiving two doses of 30 mg/m2 of fludarabine and cyclophosphamide 14.5 mcg/kg, the patient developed a neutropenic fever. Imaging revealed new ill-defined bilateral pulmonary nodules concerning for an opportunistic fungal infection, and his transplant plan was deferred. He was treated empirically with posaconazole and broad-spectrum antibiotics with the resolution of his fever. Extensive infectious workup was unrevealing. One month after treatment, his cytopenias remarkably improved to Hgb 13.9 g/dL, PLT 265,000/uL, and WBC 5.3 K/uL with ANC 4600 without G-CSF support. His performance status improved to KPS 90% and ECOG 0 with the resolution of all other symptoms while remaining off prednisone. Considering options for further treatment, the patient elected to proceed with haploidentical PBSCT. He underwent a bone marrow biopsy in preparation for HSCT two months after treatment, which demonstrated a complete response (**Figure 1**). The patient underwent a transplant with conditioning chemotherapy including 3 days of fludarabine 30 mg/m2, 2 days of cyclophosphamide 14.5 mcg/kg, and TBI with 400cGy. He achieved neutrophil engraftment on Day +22. Graft-versus-host disease (GVHD) prophylaxis consisted of cyclophosphamide on Days +3-4, mycophenolate mofetil on Days +5-35, and tacrolimus starting Day +5. Due to suspicion of acute GVHD of the gastrointestinal (GI) tract, tacrolimus was continued beyond Day +60; however, it was stopped at Day +88 with concern for drug-induced thrombotic microangiopathy. Laboratory studies were notable for elevated CH50 and soluble C5b-9 consistent with activation of the terminal complement pathway. Eculizumab was cost-prohibitive, and the patient received narsoplimab via compassionate use. His cytopenias persisted and were not fully explained by major ABO incompatibility (donor AB positive, recipient A positive), vitamin B12 deficiency (206 pg/mL), or medications. The patient received a CD34+ stem cell boost at Day +178. His course was further complicated by chronic GVHD involving the mouth, GI tract, liver, eyes, and nails. This was managed with steroids causing severe steroid myopathy and then with belumosudil with suboptimal response, eventually transitioning to ruxolitinib with no further flares of GVHD symptoms. His 1-year posttransplant bone marrow showed complete response with cytogenetic remission, 0% blasts, and 100% donor cells on FISH for chimerism.

## Methods

**Figure 2:**
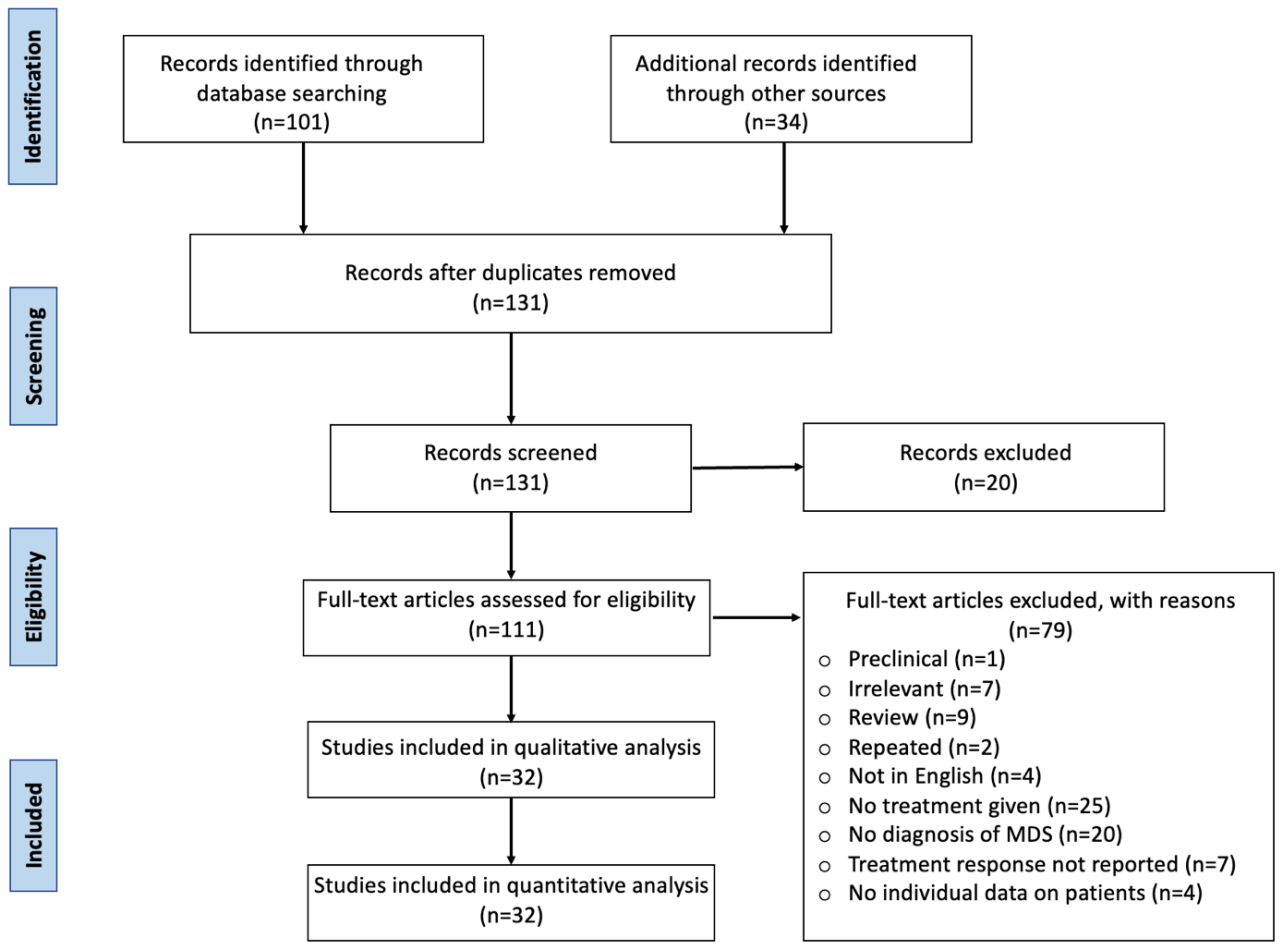
Treatment of VEXAS syndrome: systematic review PRISMA diagram.

We report a case of MDS related to VEXAS syndrome that was successfully treated with fludarabine and cyclophosphamide at the University of Kansas Medical Center. We also performed a systematic review following the Preferred Reporting Items for Systematic Reviews and Meta-Analysis guidelines (PRISMA) guidelines. A literature search was performed on 3 databases (PubMed, Cochrane, and Embase) using the MeSH terms and keywords for “VEXAS syndrome,” “myelodysplastic syndrome,” “MDS”, and “treatment for VEXAS syndrome” from the date of inception to October 2023. We screened 135 articles, and duplicates were removed. Inclusion criteria included original studies (clinical trials, retrospective, and prospective studies), case reports, and case series in all patients with a confirmed diagnosis of VEXAS syndrome with myelodysplasia or other hematological manifestations. Review articles, studies with no treatment given or no information on treatment response, studies with no individual data on patients, and studies in languages other than English, were excluded. A total of 32 studies were included for the review after primary and secondary screening (**Figure 2**). Data were extracted regarding patient sociodemographic and clinical characteristics, hematological and bone marrow findings, treatment, and treatment response as well as patient outcomes.

## Results and discussion

VEXAS syndrome is a rare autoinflammatory disease characterized by severe systemic inflammation and various clinical manifestations described by Beck et al. in 2020 (1). It has been observed that patients with VEXAS syndrome have an increased risk of developing MDS (3), which is a clonal disorder of hematopoietic stem cells that leads to ineffective blood cell production. The association between VEXAS syndrome and MDS has been reported in multiple studies, with 25% to 55% of VEXAS patients having underlying MDS (1, 3, 11, 12). Obiorah et al. showed that 10 out of 16 VEXAS patients had hematologic disorders, including MDS, multiple myeloma, monoclonal gammopathy of undetermined significance, or monoclonal B-cell lymphocytosis (6).

VEXAS syndrome is seen almost exclusively in males and is associated with older age (13); however, it has been reported in women as well (14, 15). Interestingly, those women either have monosomy X in the setting of constitutional Turner syndrome or develop an acquired X monosomy in the bone marrow karyotype, thus making them genetically similar to male patients carrying a single X chromosome with a mutation in *UBA1* gene resulting in a disease (16). Several mechanisms have been proposed alluding to interaction between *UBA1* gene and X chromosome, including X-inactivation escape by *UBA1* and skewed X-chromosome inactivation in women (17). The clinical features of VEXAS syndrome are heterogeneous and can include high-grade fever, polychondritis, skin lesions, ocular, pulmonary, and cardiac involvement. In the analyzed cohort of patients with concurrent MDS, the most prevalent features were arthritis, chondritis, or muscle involvement (71%), skin involvement (57%), fever (48%), pulmonary lesions (33%), constitutional symptoms, and thrombosis (both at 27%) (**Table 1**). 23% of patients met diagnostic criteria for relapsing polychondritis, whereas 14% of patients were diagnosed with Sweet syndrome.

**Table 1:** Characteristics of patients with VEXAS syndrome with associated MDS (n=86).

Age in years, median (range)	66 (43-83)
Gender: males/females (%)^a^	83/2 (98%/2%)
VEXAS features/systems involved: Fever, n (%) Constitutional symptoms, n (%) Thrombosis, n (%) Pulmonary lesions/airway involvement, n (%) Joint/cartilage/muscle involvement, n (%) Skin involvement, n (%) Heart involvement, n (%) Scleritis/uveitis/eye involvement, n (%) GI involvement, n (%) Lymphadenopathy, n (%) CNS/PNS involvement/neuropathy, n (%) Diagnosis of relapsing polychondritis, n (%) Diagnosis of Sweet syndrome, n (%) Reactive hemophagocytic syndrome, n (%) Small vessel/leukocytoclastic vasculitis, n (%)	** ** 41 (48%) 23 (27%) 23 (27%) 28 (33%) 61 (71%) 49 (57%) 5 (6%) 13 (15%) 7 (8%) 8 (9%) 7 (8%) 20 (23%) 12 (14%) 3 (3%) 16 (19%)
Hematological manifestations: Anemia, n (%) RBC transfusion dependency, n (%) Thrombocytopenia, n (%) PLT transfusion dependency, n (%) Leukopenia, n (%) Pancytopenia, n (%)	** ** 62 (72%) 24 (28%) 23 (27%) 4 (5%) 14 (16%) 8 (9%)
MDS subtype according to WHO 2016 classification: MDS-SLD, n (%) MDS-MLD, n (%) MDS EB 1-2, n (%) MDS-unclassifiable, n (%) MDS-RS, n (%) MDS with isolated del (5q), n (%) MDS/MPN overlap, n (%) Not reported, n (%)	** ** 6 (7%) 35 (41%) 8 (9%) 4 (5%) 6 (7%) 1 (1%) 1 (1%) 29 (34%)
MDS IPSS-R score: Very low, n (%) Low, n (%) Intermediate, n (%) High, n (%) Not reported (%)	** ** 7 (8%) 30 (35%) 12 (14%) 5 (6%) 27 (36%)
Cytogenetics: Normal, n (%) Trisomy 8, n (%) Del(20q), n (%) Other, n (%)^b^ Not reported, n (%)	** ** 27 (31%) 4 (5%) 2 (2%) 5 (6%) 53 (62%)
Somatic *UBA1* mutations: p.Met41Thr, n (%) p.Met41Val, n (%) p.Met31Leu, n (%) Other, n (%)^c^ Not reported, n (%)	** ** 31 (36%) 13 (15%) 19 (22%) 5 (6%) 18 (21%)
Other somatic mutations: DNMT3A, n (%) TET2, n (%) ZRSR2, n (%) KRAS, n (%) SMC3, n (%) PRPF8, n (%) Other, n (%)^d^	** ** 12 (14%) 9 (10%) 3 (3%) 2 (2%) 2 (2%) 2 (2%) 11 (13%)

a Gender not reported in 1 patient.

b Del(19q), Del(5q), 45, X (Turner syndrome) – each in 1 patient; -Y in 2 patients.

c c.118-1G>C (2 patients), p.ly477Ala c.1430G>C in exon 14 (1 patient), splice motif mutation (1 patient), ChrX: 47058446G→C (1 patient).

d TP53, NRAS, CBL, CALR, PPM1D, SETBP1, BRCA2, NF1, IDH1, CECR1, PRF1 (each in 1 patient).

VEXAS, Vacuoles, E1 syndrome, X-linked, autoinflammatory, somatic syndrome; MDS, myelodysplastic syndrome; GI, gastrointestinal; CNS/PNS, central nervous system/peripheral nervous system; RBC, red blood cell; PLT, platelet; WHO, world health organization; MDS-SLD, myelodysplastic syndrome with single lineage dysplasia; MDS-MLD, myelodysplastic syndrome with multilineage dysplasia; MDS EB, myelodysplastic syndrome with excess blasts; MDS-RS, myelodysplastic syndrome with ring sideroblasts; MDS/MPN, myelodysplastic syndrome/myeloproliferative neoplasm; IPSS-R, Revised International Prognostic Scoring System; UBA1, ubiquitin-like modifier activating enzyme 1.

There is a high prevalence of anemia in VEXAS patients with associated MDS (72%), with 65% of those patients having macrocytic anemia, and 39% of patients being transfusion dependent. Most patients with VEXAS syndrome develop cytopenias requiring workup with bone marrow biopsy. Examination of bone marrow aspirate in patients with VEXAS syndrome often reveals vacuolation of myeloid and erythroid precursors on bone marrow biopsy (18). Diagnosis of MDS can be challenging in the setting of inflammatory state and therapy. As pointed out by Raajimakers et al., in some patients pancytopenia and myelodysplasia are observed at a time of severe systemic inflammatory exacerbation and were not seen later in the course of the disease when symptoms improved (19).

The clinicopathological and molecular features of MDS associated with VEXAS syndrome in the analyzed cohort are consistent with the previous reports (1, 3, 5, 6). There is a higher frequency of myelodysplastic syndrome with multilineage dysplasia (MDS-MLD), low blast percentages, prevalence of IPSS-R low-risk category, and rare cases of high-risk cytogenetic abnormalities. The limitation of our analysis is a high prevalence of cases without reported MDS subtype, IPSS-R score, and cytogenetics (34%, 36%, and 62%, respectively).

Genetic variants of VEXAS-associated MDS commonly include epigenetic, splicing, and signaling factors such as DNMT3A and TET2, which were observed in 14% and 10%, respectively, which is also consistent with previous reports (19, 20). Loss of function in DNMT3A may contribute to the proinflammatory pathology of VEXAS syndrome as it has been associated with the activation of innate immune inflammatory signaling in myeloid cells (19).

A study by Ferrada et al. including 83 patients with VEXAS syndrome analyzed independent predictors of survival (21). Amino acid substitution of methionine for a valine (p.Met41Val) was associated with decreased survival compared to leucine (p.Met41Leu) and threonine (p.Met41Thr). Transfusion dependence was also associated with higher mortality, whereas ear chondritis was associated with increased survival. In the analyzed cohort, p.Met41Thr was most common (36%), followed by p.Met41Leu (22%) and p.Met41Val (15%). The subtype of *UBA1* mutation was not reported in one-fifth of cases.

The treatment of VEXAS syndrome and associated MDS is challenging and not yet well-defined (22). However, there have been some treatment strategies that have shown promise. Diarra et al. reported successful allogeneic hematopoietic stem cell transplantation in patients with VEXAS syndrome and severe inflammatory symptoms or MDS (23). Another treatment option is the use of DNA hypomethylating agents such as azacitidine, which has shown efficacy in VEXAS patients with MDS (15, 19, 22, 24–27). In a French registry, clinical responses were observed in 46% of VEXAS patients with MDS after treatment with azacitidine (22). Other potential treatments include anti-IL6 monoclonal antibodies such as tocilizumab (12, 28–31), anti-IL1 receptor antagonists such as anakinra (25, 30, 32), and Janus kinase (JAK) inhibitors including ruxolitinib and others (33–37).

In the analyzed cohort of patients with VEXAS and MDS, almost all patients received corticosteroids (CS) at some point in their disease course (98%), with favorable responses seen in the majority of those patients (94%) (**Table 2**). Steroid-sparing agents have been used with varying degrees of success: none of the patients achieved a complete response, and most patients required concomitant steroids or combinations with other agents such as rituximab or intravenous immunoglobulin (IVIG). A subset of patients has been exposed to biologic agents targeting IL1-R, IL-6R, IL17, and other, or tumor necrosis factor (TNF) alpha inhibitors, with some patients achieving complete response when those agents were combined with CS. A similar response was seen with JAK inhibitors such as filgotinib, tofacitinib, and upadacitinib. Localized skin reactions were observed with administration of anakinra and tocilizumab (30, 36, 38).

**Table 2:** Treatment responses in patients with VEXAS syndrome and MDS (n=86).

Class of medication, number of patients treated (%)	Best response to treatment	References
**1. Corticosteroids (CS), 84 (98%)**	Favorable response in 94%^a^ but unable to wean off steroids	(12, 15, 19, 20, 22–49)
**2. Steroid-sparing agents/DMARDs:** **a. Methotrexate (MTX), 27 (31%)** ** b. Azathioprine, 22 (26%)** ** c. Cyclosporine, 14 (16%)** ** d. Cyclophosphamide (CYC), 7 (8%)** ** e. Hydroxychloroquine (HCQ), 8 (9%)** ** f. Mycophenolate mofetil (MMF), 14 (16%)** ** g. Thalidomide, 2 (2%)** ** h. Leflunomide, 3 (3%)** ** i. Tacrolimus, 1 (1%)** ** j. Dapsone/dusilone, 9 (10%)** ** k. Colchicine, 7 (8%)** ** l. Salazopirine, 1 (1%)**	PR in combination with other agents, durable response with CS PR with decreased CS dose PR in combination with CS Refractory unless in combination with rituximab PR in combination with dapsone or CS PR in combination with rituximab or IVIG No response PR Limited response PR in combination with HCQ No response No response	(12, 15, 22, 23, 25, 26, 30, 32–34, 36–42, 44, 47–49) (12, 15, 19, 22, 23, 26, 28, 30, 34–37, 41, 47–49) (12, 15, 25, 28, 30, 32, 33, 42, 43, 49) (22, 28, 30, 31, 36, 47, 48) (22, 23, 26, 33, 41, 47) (12, 19, 22, 23, 30, 31, 34, 36–38, 40, 47) (33, 41) (25, 39, 47) (28) (22, 23, 28, 30, 47) (23, 26, 40, 43, 45, 49) (22)
**3. Biologics:** **a. Adalimumab, 9 (10%)** ** b. Anakinra, 20 (23%)** ** c. Canakinumab, 6 (7%)** ** d. Situximab, 1 (1%)** ** e. Infliximab, 9 (10%)** ** f. Ustekinumab, 3 (3%)** ** g. Etanercept, 3 (3%)** ** h. Abatacept, 1 (1%)** ** i. Anti-IL1R, 5 (6%)** ** j. Anti-IL6R, 6 (7%)** ** k. Low dose IL-2, 1 (1%)** ** l. Secukinumab (anti-IL17), 1 (1%)** ** m. TNF inhibitor, 4 (5%)** ** n. Tocilizumab, 16 (19%)** ** o. IVIG, 7 (8%)** ** p. Rituximab, 8 (9%)**	PR alone or in combination with MTX CR in combination with CS No response No response No response No response SD in combination with CS No response PR with decreased CS dose PR with decreased CS dose PR with decreased CS dose SR in combination with IVIG No response CR in combination with CS PR in combination with anti-IL17 Refractory unless in combination with CYC or MMF	(12, 23, 26, 30, 32, 37, 38, 48) (19, 23, 25, 26, 30, 32, 33, 36, 45, 47, 48) (23, 30, 32) (23) (25, 26, 30, 32, 33, 42, 47, 48) (26, 33, 48) (15, 37, 49) (47) (22, 41) (22, 41) (41) (48) (22, 41) (12, 23, 25, 28–31, 37, 38, 40, 45, 47–49) (19, 23, 25, 30, 36, 47, 48) (12, 30, 31, 36, 37, 47)
**4. JAK inhibitors:** **a. Tofacitinib, 3 (3%)** ** b. Ruxolitinib, 4 (5%)** ** c. Baricitinib, 4 (5%)** ** d. Filgotinib, 1 (1%)** ** e. Upadacitinib, 1 (1%)**	CR Improvement in systemic manifestations only No response CR in combination with CS CR in combination with CS	(32, 34, 42) (12, 23, 33) (33, 37, 45, 48) (35) (36)
**5. MDS-directed therapy:** **a. Erythropoietin/darbepoetin/ESA, 7 (8%)** ** b. Azacitidine, 35 (41%)**	SD CR	(15, 19, 30, 32, 33, 39) (12, 15, 19, 20, 22–27, 30, 32, 38, 41)
**6. Other:** **a. Apremilast, 1 (1%)** ** b. Tranilast** ** c. Erythromycin** ** d. Fludarabine**	No response PR in combination with CS Limited to no response CR in combination with CYC	(26) (24) (28) Reported case
**7. Allo-HSCT, 13 (16%)**	CR	(12, 22, 23, 33, 42, 45, 46)

^a^Represents row percentage.

VEXAS, Vacuoles, E1 syndrome, X-linked, autoinflammatory, somatic syndrome; MDS, myelodysplastic syndrome; DMARDs, disease-modifying antirheumatic drugs; PR, partial response; CR, complete response; SD, stable disease; IVIG, intravenous immunoglobulin; IL, interleukin; TNF, Tumor Necrosis Factor; JAK, Janus kinase, ESA, erythropoietin-stimulating agent; Allo-HSCT, Allogeneic hematopoietic stem cell transplantation.

MDS-directed therapies such as erythropoietin-stimulating agents were attempted in 8% of patients with stable disease as the best response, while hypomethylating agents (HMA) such as azacitidine were used in 41% of patients with high efficacy. In the French VEXAS cohort, azacitidine was effective in 46% of patients (22), which supports the hypothesis that azacitidine may control steroid-dependent inflammatory and autoimmune disorders (50). While allogeneic HSCT is a curative option for VEXAS syndrome that is refractory to immunosuppression and cytokine-inhibiting agents, it is a high-risk treatment modality with associated mortality requiring careful selection of patients. There is currently no evidence guiding the selection of patients with VEXAS syndrome who will benefit from HSCT. Ongoing phase II trial of allogeneic HSCT for subjects with VEXAS syndrome (NCT05027945) may shed light on these guidelines. The development of the Autoinflammatory Disease Alliance (AIDA) registry for patients with VEXAS syndrome will also provide valuable real-world evidence for understanding the natural history of the disease and guiding therapeutic approaches (NCT05200715).

In the presented case, we observed complete resolution of both systemic and hematological manifestations of VEXAS syndrome and associated MDS after administration of 2 doses of conditioning regimen with fludarabine and cyclophosphamide. This was further confirmed by repeat bone marrow biopsy with the disappearance of vacuoles in both myeloid and erythroid precursors. The patient elected to proceed with HSCT as per the previous plan of treatment and is currently beyond 6 months after transplant with complications including pancytopenia requiring stem cell boost and chronic graft-versus-host disease. To our knowledge, this is the first case reporting a complete response using fludarabine and cyclophosphamide in VEXAS syndrome. Further research is needed to better understand the pathogenesis and optimal management of VEXAS syndrome.

## Data availability statement

The original contributions presented in the study are included in the article/supplementary material. Further inquiries can be directed to the corresponding author.

## Ethics statement

Written informed consent was obtained from the individual(s) for the publication of any potentially identifiable images or data included in this article.

## Author contributions

PB: Writing – original draft. JG: Writing – original draft. MS: Writing – original draft. MA: Writing – original draft. MK: Writing – review & editing. NS: Writing – review & editing. NA: Writing – review & editing. AS: Writing – review & editing. AY: Writing – review & editing. DZ: Data curation, Visualization, Writing – review & editing. JPM: Writing – review & editing. MM: Data curation, Writing – original draft, Writing – review & editing.
